# Cardiogenic Shock in a Patient With 4G/4G PAI Polymorphism and MTHFR A1298C Mutation

**DOI:** 10.7759/cureus.53554

**Published:** 2024-02-04

**Authors:** Ijeoma Orabueze, Inemesit Akpan, Valerie Cluzet, Mark Harrison

**Affiliations:** 1 Internal Medicine, Vassar Brothers Medical Center, Poughkeepsie, USA; 2 Internal Medicine, Piedmont Athens Regional, Athens, USA; 3 Infectious Diseases, Vassar Brothers Medical Center, Poughkeepsie, USA

**Keywords:** hyperhomocysteinemia, pai polymorphism, 4g/4g pai, mthfr, cardiogenic shock

## Abstract

Myocardial infarction (MI) remains a common cause of morbidity and mortality. Although many well-known risk factors exist, the association between inherited thrombophilia disorders and acute MI is not well described. Here, we present a case of a 75-year-old male with known 4G/4G PAI-1 polymorphism, methylenetetrahydrofolate reductase (MTHFR) mutation, and peripheral artery disease (PAD) post stent placement who presented with cardiogenic shock in the setting of acute MI with no prior significant cardiac history.

## Introduction

Regardless of A1298C or C677T involvement, the methylenetetrahydrofolate reductase (MTHFR) gene mutation decreases MTHFR activity. A homozygous mutation is associated with higher homocysteine levels, and a heterozygous mutation with mildly raised levels compared to the lack of the mutation. Hyperhomocysteinemia is a risk factor for various cardiovascular diseases and is becoming more significant because of its impact on patient morbidity and mortality [1,2]. Also, plasminogen activator inhibitor type 1 (PAI-1) is the main inhibitor of fibrinolysis, and high levels have been associated with an increased risk of cardiovascular disease. It was reported that the 4G/5G and 4G/4G genotypes have been associated with higher gene expression and higher PAI-1 levels in the circulation, resulting in an increased risk for thrombotic events such as myocardial infarction (MI) [3,4]. One meta-analysis found a 20% increased risk of MI in patients with the 4G/4G genotype [3]. The combination of both MTHFR gene mutation and PAI-4G/4G polymorphism, together with interacting environmental and non-modifiable risk factors, led to a lethal presentation of cardiogenic shock after a non-ST elevated MI in our patient [3,4]. We highlight this case to educate on various presentations of coronary artery disease (CAD) and highlight the role of genetic mutations in MI. 

## Case presentation

**Figure 1:**
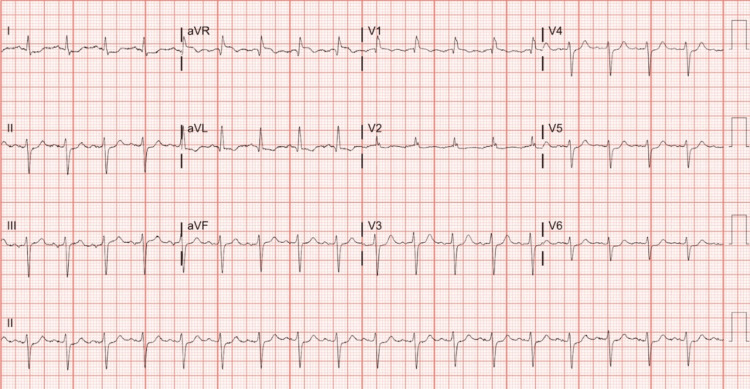
The EKG on presentation with sinus tachycardia, LAFB, and RBBB LAFB: Left anterior fascicular block; RBBB: Right bundle branch block

A 75-year-old male with a medical history of 4G/4G PAI polymorphism and MTHFR mutation at A1298C, hypertension, and peripheral artery disease (PAD) status post stent placement 10 months prior, maintained on low-dose acetylsalicylic acid (ASA) and clopidogrel presented to the hospital with one week of melanic stool, substernal chest pain, and dysphagia. On presentation, he was hemodynamically unstable with a troponin of 2664 ng/l (Table 1) and hemoglobin of 7.6 g/dl (Table 1), requiring two pressors and two units of packed RBCs (PRBCs). His initial EKG (Figure 1) showed sinus tachycardia with the right bundle branch and left anterior fascicular block. Upper gastrointestinal (GI) endoscopy revealed a non-bleeding duodenal ulcer and diffusely erythematous gastric body and gastric antrum. Transthoracic echocardiogram (TTE) demonstrated an ejection fraction (EF) of 15-20% with severe global hypokinesis and severely reduced right ventricular systolic function. 

**Table 1:** This table shows the Hgb, troponin, WCC, and lactate trends over 48 hours. Troponin in ng/l and the normal range is <=22 ng/l (nanogram per liter). The unit of hemoglobin (Hgb) is in g/dl, and the normal range is between 13.5-17.0 g/dl (grams per decilitre). For white cell count (WCC), the normal range is 3.5-10*10(9)/l. Lactate unit is in mmol/l is millimole per liter. Normal range is 0.5-2.2 mmol/l.

Laboratory values	Day 0	Day 1	Day 2
Hgb	7.6 g/dl	9.3 g/dl	9.7 g/dl
Troponin	2664 ng/l	3135 ng/l	2983 ng/l
WCC	24.4 *10 (9)/L	21.9 *10 (9)/L	20.3 *10 (9)/L
Lactate	3.2 mmol/l	2.2 mmol/l	2.1 mmol/l

**Figure 2:**
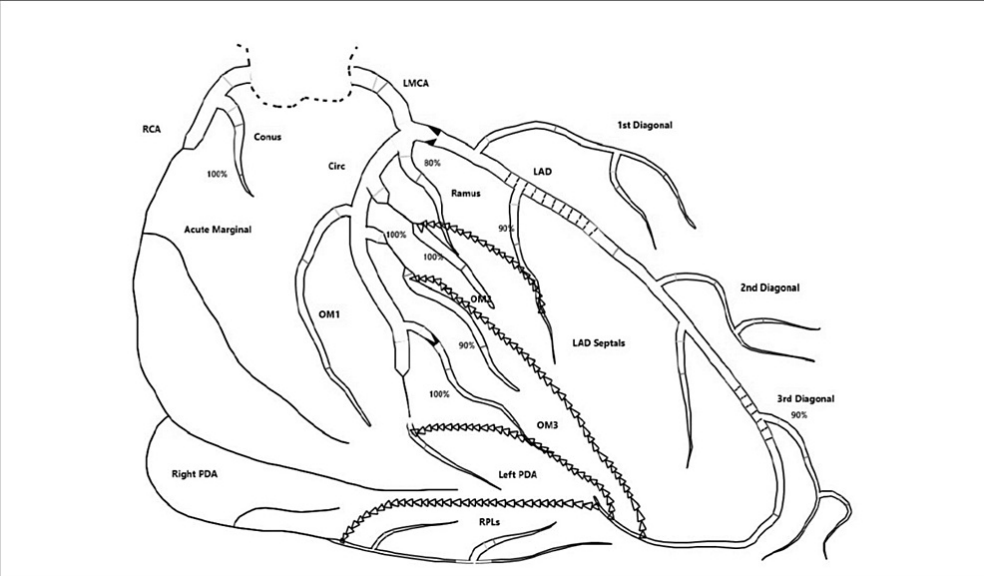
The left heart catheterization revealed no angiographically significant disease in the left main coronary artery, a discrete 80% ostial lesion in the LAD artery, a diffuse 90% mid lesion in the LAD artery, a diffuse 90% proximal lesion in the LAD artery, a discrete 100% distal lesion in circumflex, a discrete 100% proximal lesion in the MARG1, a discrete 100% proximal lesion in the second OM artery, a discrete 100% proximal lesion in the MARG1, a discrete 100% proximal lesion in the RCA, a discrete 100% proximal lesion in the MARG1, collateral flow from the LAD artery to the right PDA, collateral flow from the LAD artery to the left PDA, collateral flow from the LAD artery to the second OM artery, collateral flow from the LAD septal to the MARG1, ventriculography: EF 15%. The final recommendations were for CABG. LAD: left anterior descending; MARG1: marginal artery; OM: Obtuse marginal; RCA: Right coronary artery; PDA: posterior descending artery; EF: ejection fraction; CABG: coronary artery bypass graft

Left and right heart cardiac catheterization (Figure 2) (Video 1) was performed, and the patient was found to have severe three-vessel CAD involving the left anterior descending, left circumflex, and right coronary arteries. Cardiovascular Surgery was consulted for coronary artery bypass grafting (CABG). A bilateral carotid Doppler showed no significant disease. Extensive discussions were undertaken with Interventional Cardiology, Cardiothoracic Surgery, the patient, and his family; it was deemed that his diffuse, extensive multivessel coronary atherosclerosis was not amenable to percutaneous coronary intervention (PCI) or to coronary artery bypass surgery. He was offered the option of cardiac transplant and referral to a quaternary care center for this, along with consideration for possible mechanical circulatory support with a left ventricular assist device (LVAD). The patient sought a second opinion at another hospital, where he underwent a PCI to his left anterior descending artery (LAD). Upon follow-up at our heart failure bridge clinic, he continued to report dyspnea with exertion, but there were no anginal symptoms. He was managed on guideline-directed medical therapy with Entresto and carvedilol, and a repeat TTE nine months later showed an improved EF of 40-49%. 

**Video 1:** The left heart catheterization revealed no angiographically significant disease in the left main coronary artery, a discrete 80% ostial lesion in the LAD artery, a diffuse 90% mid lesion in the LAD artery, a diffuse 90% proximal lesion in the LAD artery, a discrete 100% distal lesion in circumflex, a discrete 100% proximal lesion in the MARG1, a discrete 100% proximal lesion in the second OM artery, a discrete 100% proximal lesion in the MARG1, a discrete 100% proximal lesion in the RCA, a discrete 100% proximal lesion in the MARG1, collateral flow from the LAD artery to the right PDA, collateral flow from the LAD artery to the left PDA, collateral flow from the LAD artery to the second OM artery, collateral flow from the LAD septal to the MARG1, ventriculography: EF 15%. The final recommendations were for CABG. LAD: left anterior descending; MARG1: marginal artery; OM: Obtuse marginal; RCA: Right coronary artery; PDA: posterior descending artery; EF: ejection fraction; CABG: coronary artery bypass graft

## Discussion

Cardiovascular disease remains the most common cause of death in the United States [5]. Although we know metabolic syndromes, age, genetics, gender, lifestyle, and environment are classic risk factors, the development of MI can only be partially ascribed to them [5]. Another factor that has not been sufficiently studied is PAI-1 activity and its polymorphisms, as well as the coexistence of the PAI polymorphisms with MTHFR mutation. 

Homocystinuria is a rare genetic condition where a defective enzyme causes homocysteine to accumulate to high levels in the blood [6]. An inherited mutation in the MTHFR gene is one of the common causes of homocysteinemia [6]. There is a strong association between mutations in MTHFR, prothrombin 20210A, and the 4G polymorphism of the PAI-1 and an increased risk of thrombosis [6]. 

A study by Gogu et al. on patients with cerebral venous thrombosis noted a coexistence of MTHFR mutations with PAI 4G/5G mutations. The study reported that MTHFR mutations were associated with higher homocysteine, low-density lipoprotein cholesterol (LDLc), and highly sensitive C-reactive protein levels and that hyperhomocysteinemia was a risk factor for cardiovascular and cerebrovascular diseases [7]. Hyperhomocysteinemia is believed to cause endothelial cell damage, reduce vessel flexibility, and alter the process of hemostasis [8]. It could also enhance the adverse effects of the risk factors for CAD, such as hypertension, smoking, and lipid and lipoprotein metabolism, as well as promote the development of inflammation [8]. 

On the other hand, PAI-1, a single-chain glycoprotein, is a member of the superfamily of serine-protease inhibitors. It inhibits fibrinolysis by inhibiting the conversion of plasminogen to plasmin, specifically by inhibiting two main plasminogen activators: urokinase-type plasminogen activator and tissue-type plasminogen activator. The PAI-1 stabilizes the thrombus formed during injury, allowing wound healing. These glycoproteins are typically secreted by endothelial cells but stored and released from platelets [9,10]. Several studies have demonstrated an increased level of PAI-1 messenger RNA (mRNA) in severely atherosclerotic vessels as compared to normal or mildly affected vessels, indicating that there could be an association between polymorphism and the development of CAD [9]. Moreover, it has been reported that patients with the 4G/4G polymorphism have higher levels of the PAI protein than the 4G/5G polymorphism. A study by Eriksson et al. on patients 45 years of age and younger who had developed MI revealed that patients with higher levels of PAI-1 activity in the serum had higher numbers of 4G alleles [11]. They concluded that there was a strong association between the 4G/5G polymorphism in the PAI-1 promoter and the development of MI, with evidence of an independent etiologic role. Although the PAI 4G/5G genotype and its relation to the risk of thrombosis is the most studied, our patient has the 4G/4G genotype, which has also been associated with higher protein levels [10,12]. 

Our patient received his diagnosis of PAI-1 polymorphism and MTHFR A1298C mutation 10 years before this presentation, when he presented with a clot in his finger and underwent evaluation for thrombophilia. He was then placed on high-dose aspirin. However, several years later, he also developed severe PAD, necessitating stent placement. It is possible that the coexistence of both mutations increased his risk of PAD and CAD. Although acute anemia secondary to GI bleed can lead to type II MI, the presence of certain genetic mutations like in our patient can predispose to severe CAD requiring intervention. More studies are needed to establish the true risk of cardiovascular disease associated with this gene-gene interaction so that early measures can be taken to prevent these outcomes. So far, physical activity, along with medications such as thiazolidinediones (which have beneficial effects on the fibrinolytic system, especially on PAI-1 levels) and statins, have been recommended for management in patients with PAI-1 polymorphism [9]. Our patient was already on a statin. More research is also needed to inform which medications or lifestyle interventions may reduce the risk of CAD and PAD in patients with these genetic polymorphisms.

## Conclusions

The genetically determined risk of CAD is based on multiple gene-gene interactions and environmental risk factors. Here, we argue that the coexistence of the MTHFR mutation, which is a risk factor for coronary heart disease, peripheral vascular disease, and cerebrovascular diseases, together with the PAI-1 homozygous polymorphism, contributed to the patient's presentation.

The clinical evidence of a cause-and-effect relationship between PAI-1 gene polymorphism and CAD is still limited. It is, therefore, imperative that more studies are performed to establish the relationship and discover drugs that can be used to slow down the progression to PAD or CAD. 
